# Reconstruction of the Metabolic Potential of Acidophilic *Sideroxydans* Strains from the Metagenome of an Microaerophilic Enrichment Culture of Acidophilic Iron-Oxidizing Bacteria from a Pilot Plant for the Treatment of Acid Mine Drainage Reveals Metabolic Versatility and Adaptation to Life at Low pH

**DOI:** 10.3389/fmicb.2016.02082

**Published:** 2016-12-22

**Authors:** Martin Mühling, Anja Poehlein, Anna Stuhr, Matthias Voitel, Rolf Daniel, Michael Schlömann

**Affiliations:** ^1^Institute of Biological Sciences, Technische Universität Bergakademie FreibergFreiberg, Germany; ^2^Georg-August-University Göttingen, Genomic and Applied Microbiology and Göttingen Genomics, LaboratoryGöttingen, Germany

**Keywords:** acid mine drainage, metagenomics, iron oxidation, microaerophilic bacteria, *Gallionella*, *Sideroxydans*, pH homeostasis, cyanophycin

## Abstract

Bacterial community analyses of samples from a pilot plant for the treatment of acid mine drainage (AMD) from the lignite-mining district in Lusatia (East Germany) had previously demonstrated the dominance of two groups of acidophilic iron oxidizers: the novel candidate genus “*Ferrovum*” and a group comprising *Gallionella*-like strains. Since pure culture had proven difficult, previous studies have used genome analyses of co-cultures consisting of “*Ferrovum*” and a strain of the heterotrophic acidophile *Acidiphilium* in order to obtain insight into the life style of these novel bacteria. Here we report on attempts to undertake a similar study on *Gallionella*-like acidophiles from AMD. Isolates belonging to the family *Gallionellaceae* are still restricted to the microaerophilic and neutrophilic iron oxidizers *Sideroxydans* and *Gallionella*. Availability of genomic or metagenomic sequence data of acidophilic strains of these genera should, therefore, be relevant for defining adaptive strategies in pH homeostasis. This is particularly the case since complete genome sequences of the neutrophilic strains *G. capsiferriformans* ES-2 and *S. lithotrophicus* ES-1 permit the direct comparison of the metabolic capacity of neutrophilic and acidophilic members of the same genus and, thus, the detection of biochemical features that are specific to acidophilic strains to support life under acidic conditions. Isolation attempts undertaken in this study resulted in the microaerophilic enrichment culture ADE-12-1 which, based on 16S rRNA gene sequence analysis, consisted of at least three to four distinct *Gallionellaceae* strains that appear to be closely related to the neutrophilic iron oxidizer *S. lithotrophicus* ES-1. Key hypotheses inferred from the metabolic reconstruction of the metagenomic sequence data of these acidophilic *Sideroxydans* strains include the putative role of urea hydrolysis, formate oxidation and cyanophycin decarboxylation in pH homeostasis.

## Introduction

The lignite-mining district in Lusatia (Germany) is rich in pyrite and marcasite. Mining activities, therefore, cause a dramatic increase in pyrite/marcasite surface exposure and subsequent oxidative processes which, in turn, result in acidic waters with high sulfate and ferrous iron loads. Remediation of these acidic waters is required in order to avoid environmental damage following drainage from active and abandoned mines. Key to the unceasing formation of these acid mine drainage (AMD) waters is the continuous oxidation of ferrous iron to ferric iron which itself is the main oxidant in this process. Acidophilic iron-oxidizing bacteria that gain energy for their metabolic activities from the oxidation of ferrous iron to ferric iron at low pH play a pivotal part, because their activity ensures sustained ferrous iron oxidation at pH levels where ferrous iron becomes stable even in the presence of oxygen.

**Figure 1:**
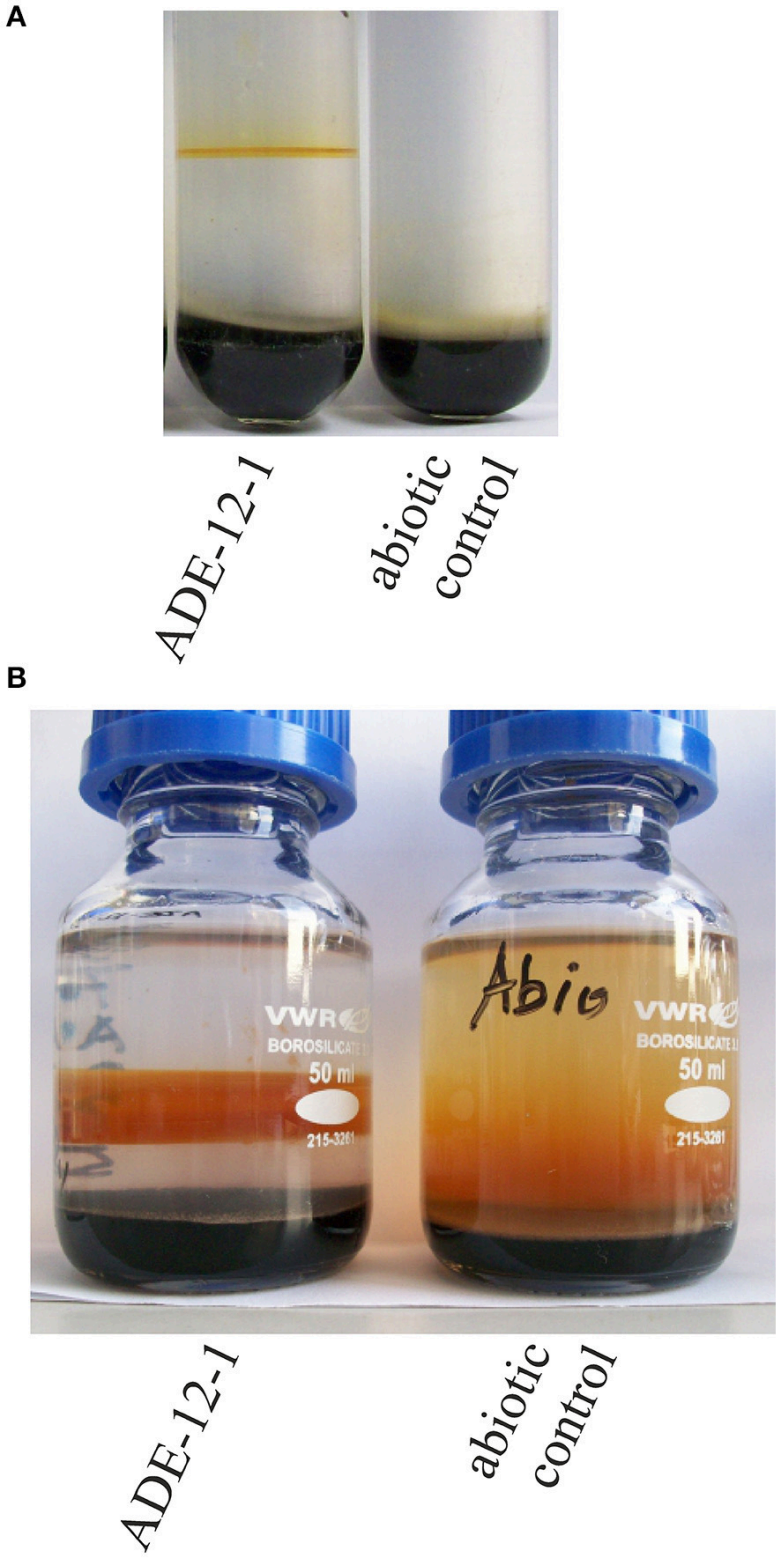
**Photographs of enrichment culture ADE-12-1 in gradient tube (A)** and as 50-mL volume gradient culture **(B)**. Photographs were taken after 14 days **(A)** or 35 days **(B)** of incubation.

Although, acidophilic iron-oxidizing bacteria are largely responsible for the generation of AMD, they can also contribute to the reduction of its iron and of some of its sulfate load *via* ferrous iron oxidation with subsequent precipitation of ferric iron hydroxysulfate minerals (Janneck et al., 2010). An example of such a biotechnological process is provided by the treatment plant Tzschelln (Janneck et al., 2010), a 10-qm^3^ pilot-scale operation (Figure 1) for the bioremediation of AMD water from the open-pit lignite mine Nochten (Lusatia, Germany). This process involves, in essence, the aeration of AMD water and subsequent ferrous iron oxidation by acidophilic iron-oxidizing microorganisms. The resulting ferric iron then precipitates as the amorphous iron hydroxy sulfate mineral schwertmannite (Fe_16_[O_16_|(OH)_10_|(SO_4_)_3_] • 10 H_2_O; Bigham et al., 1996), because the average hydraulic retention time (8 h) of the AMD water within the treatment plant ensures that the pH is maintained at approximately 3 (pH 2.85–3.1). Schwertmannite has various applications as a pigment or as a sorbent for the removal of arsenic from aqueous solutions (Janneck et al., 2010).

Since the microbial community within this treatment plant is likely to play a fundamental role in the performance of the biotechnological process, it has been investigated in a series of studies covering almost 10 years. Employing culture independent molecular techniques, surveys of the bacterial diversity within the treatment plant Tzschelln previously revealed the dominance of two bacterial groups: strains belonging to the novel putative genus “*Ferrovum*” (Johnson et al., 2014) and strains that are, based on their 16S rRNA gene sequences, related to neutrophilic iron-oxidizing strains of the genus *Gallionella* (Heinzel et al., 2009a,b). *Acidithiobacillus* strains were also detected in the AMD entering the treatment plant, though these only played a minor role within the oxidation basin of the treatment plant (Heinzel et al., 2009a). Moreover, the same observations were made following the commissioning of a new pilot plant where “*Ferrovum*” and *Gallionella*-like strains also dominated the bacterial diversity throughout an annual cycle (Heinzel et al., 2009b). Both bacterial groups belong to the *Betaproteobacteria* and, therefore, represent the first acidophilic iron oxidizers within this phylogenetic class (Hallberg et al., 2006; Mosler et al., 2013; Johnson et al., 2014; Ullrich et al., 2016a,b). At that time, the genus *Gallionella* was only known to comprise iron oxidizing strains that occur in circumneutral and microaerobic environments rich in ferrous iron (e.g., Hanert, 1968). In contrast to this, much less was known of the genus “*Ferrovum*” which was newly proposed to accommodate streamer forming acidophilic iron oxidizing strains with an autotrophic life style (e.g., Hallberg et al., 2006; Johnson et al., 2014).

Attempts to isolate “*Ferrovum*” strains or representatives of the acidophilic *Gallionella* relatives were initially based on the use of the overlay-plate technique in combination with the iFe medium (e.g., Johnson et al., 2014), which is a modified version of a medium developed for a broad range of acidophilic iron oxidizers (Johnson and McGinness, 1991; Johnson and Hallberg, 2007). However, the subsequent development and use of a novel medium (Artificial Pilot Plant Water – APPW – medium) that simulates the chemical composition of the water within the treatment plant resulted in cultures composed of two strains, a “*Ferrovum*” and an *Acidiphilium* strain (Tischler et al., 2013; Ullrich et al., 2015). All attempts to obtain clonal cultures of “*Ferrovum*” strains from these mixed cultures failed, though this did not prevent detailed genome analyses of three “*Ferrovum*” strains (Ullrich et al., 2016a,b). The presence of *Acidiphilium* in any of the “*Ferrovum*”-containing cultures may partially be explained by hydrolysis of agar or agarose (used as solidifying agent) at pH 3 which results in the release of organic acids that seem to be toxic to “*Ferrovum*.” The heterotrophic *Acidiphilium* contaminant is capable of oxidizing these organic acids to carbon dioxide and, by doing so, provides an improved environment for growth of “*Ferrovum*” (Ullrich et al., 2015). This also indicates that *Acidiphilium* in the lower layer of the overlay-plate (Johnson and McGinness, 1991; Johnson and Hallberg, 2007) was in these cases insufficient to remove all of the organic molecules from the top layer where the samples were plated on. Once present within a “*Ferrovum*” colony on solidified media, *Acidiphilium* can apparently not easily be removed in subsequent efforts that merely favor growth of the iron oxidizers in the mixed culture, an observation that has also been made by others (Johnson et al., 2014).

Using the same approach the isolation of the acidophilic *Gallionella* relatives proved even more difficult and did not even result in a mixed culture, though colonies on overlay-plates screened by PCR with *Gallionella*-specific primers indicated in some instances their presence (Gelhaar, 2012). Interestingly, comparative media tests also showed that most colonies harboring acidophilic *Gallionella* relatives were obtained with a modified version of the APPW medium in which the phosphate concentration was adapted to that of the iFe medium (APPW-PO4 medium: Tischler et al., 2013). Whether phosphate addition is the most important trigger for improved recovery of acidophilic *Gallionella* relatives remains to be determined since an overall reduction of the number of colonies, in particular those comprising *Acidithiobacillus* strains, may indicate potential indirect effects.

Nevertheless, all subsequent enrichment campaigns for *Gallionella* relatives were built on the use of APPW-PO4, but—assuming a microaerophilic life style similar to that of the neutrophilic iron oxidizer *Gallionella ferruginea*—in combination with the gradient tube technique developed by Kucera and Wolfe (1957) for the isolation of neutrophilic iron-oxidizing *Gallionella* strains. Additionally, the pH of the APPW-PO4 was adjusted to 3.5 in order to simulate the acidic pH of the AMD within treatment plant Tzschelln. Given the difficulties with respect to isolation of clonal cultures, we aimed to employ a metagenomic approach for the analysis of such an enrichment culture followed by subsequent reconstruction of metabolic features of acidophilic *Gallionellaceae* strains.

## Materials and methods

### Origin of samples and enrichment of microaerophilic strains

The AMD sample used to obtain enrichment cultures was collected on 19 March 2014 from the inflow into the treatment plant Tzschelln. A 100-μL aliquot of the AMD was used to inoculate gradient tubes (see below) on 20 March 2014.

Enrichment of microaerophilic and acidophilic iron-oxidizing microorganisms was achieved using gradient tubes of semi-solid APPW-PO4 and incubation in microaerobic chambers (2.5 L Anaerojar with Campygen pads, OXOID). This approach was based on the assumption that the acidophilic *Gallionella*-like strains are physiologically similar to neutrophilic *Gallionella* which have long been known to occur mainly under ferrous iron rich and oxygen limiting conditions (Engel and Hanert, 1967). Gradient tubes originally developed by Kucera and Wolfe (1957) were produced by encapsulating iron sulfide (prepared according to Emerson and Floyd, 2005) within agarose (0.5% w/v) at the bottom of a glass tube, with a semi-solid [0.15% (w/v) agarose] layer of APPW-PO4 medium (pH 3.5) atop. Tests had shown that this setup led to better results than those using, for instance, iron carbonate as source of ferrous iron (data not shown). Additionally, a semisolid layer proved also to be superior for the isolation of microaerophilic enrichment cultures in comparison to a liquid layer of APPW-PO4 medium, similar to what has been suggested by Hallbeck et al. (1993). The pH within the semi-solid medium in uninoculated control tubes was found to be constant. Although, the pH was not determined within enrichment culture ADE-12-1 we believe that it did not increase since precipitation of ferric iron results in the release of protons.

### Analysis of the bacterial diversity

Genomic DNA extraction from the microaerophilic enrichment culture ADE-12-1 was carried out using the PowerSoil DNA Isolation Kit (MoBio). PCR amplification of approx. 1300-bp 16S rRNA gene fragments was achieved using primers 27f (5′-AGAGTTTGATCCTGGCTCA) and 1387r (5′-GGGCGG(AT)GTGTACAAGGC) and a cycle protocol consisting of an initial denaturing step at 95°C for 5 min followed by 30 cycles (95°C for 30 s, 55°C for 30 s, 72°C for 90 s) and a final extension step at 72°C for 5 min. PCRs were carried out in 25-μL volumes containing 20 μmol L^−1^ of each of the two primers, 1.5 mmol L ^−1^ MgCl_2_, 8 μmol L^−1^ of each of the dNTPs, 2 U of DreamTaq DNA polymerase and 15 ng of genomic DNA as template for amplification. The PCR amplicons of two independent PCRs were combined in order to limit the impact of potential differences between the setups for the PCRs. PCR amplicons were subsequently purified using the Ultra Clean PCR Clean-Up Kit (MoBio) and ligated into the pSC-A-amp/kan Vektor using the StrataClone PCR Cloning Kit (Stratagene) according to the manufacturer's protocol. These constructs were finally transformed into the StrataClone SoloPack competent cells (Stratagene). 100 clones were screened by amplified ribosomal DNA restriction analysis (ARDRA). In brief, 4 μL of a PCR product obtained with vector primers T3 and T7 were digested in a 10-μL reaction with 1 U of restriction endonuclease BsuRI (Fermentas). The nucleotide sequence of a total of 30 clones representing the 18 observed restriction patterns were subsequently determined using Sanger sequencing (Eurofins Genomics, Germany).

Alignment of the 16S rRNA gene sequences and amino acid sequences was performed within ARB (Ludwig et al., 2004) against the SILVA database (Pruesse et al., 2007) or within MEGA 6 (Tamura et al., 2013), respectively. Phylogenetic analyses were carried out using the neignor-joining approach within MEGA 6 (Tamura et al., 2013).

16S-tag amplicons for subsequent Illumina sequence analysis were obtained according to Illumina's 16S Metagenomic Sequencing Library Preparation protocol which aims at PCR amplification of the V3-V4 region of the 16S rRNA gene using the PCR primers recommended by Klindworth et al. (2013). However, instead of an aliquot of the genomic DNA preparations an approx. 1300-bp PCR product (obtained with primers 27f/1387r) was used as template for the index PCR during Nextera library preparation. This was necessary since direct amplification of the V3-V4 region from genomic DNA using the PCR primers of Klindworth et al. (2013) did not result in any PCR product. Although, this represents a nested PCR approach, it should still permit a reliable comparison of the results with those from the sequence analysis of the clone library of 16S rRNA gene fragments which was based on the same 1300-bp PCR amplicons (see above).

16S-tag Illumina datasets were processed with Usearch version 8.1.1861 (Edgar, 2010). Paired-end reads were truncated to 250 bp to remove low-quality sequence tails, subsequently merged and quality-filtered. Filtering included the removal of low quality reads (maximum number of expected errors >1 and more than 1 ambiguous base). Processed sequences of all samples were joined and clustered into operational taxonomic units (OTUs) at 3% genetic divergence using the UPARSE algorithm implemented in Usearch. Chimeric sequences were identified *de novo* during clustering and removed together with singletons (OTUs consisting of only one sequence). Afterwards, putative chimeric sequences were removed using the Uchime algorithm implemented in Usearch in reference mode with the most recent RDP training set (version 15) as reference dataset (Cole et al., 2009). Afterwards, OTU sequences were taxonomically classified using a BLAST alignment against the most recent SILVA database (SILVA SSURef 123 NR) (Quast et al., 2013). All non-bacterial OTUs were removed based on their taxonomic classification in the database. Subsequently, processed sequences were mapped to OTU sequences to calculate the distribution and abundance of each OTU in every sample.

### Metagenome sequencing, assembly and annotation

Metagenome sequencing of the microaerophilic enrichment culture ADE-12-1 was performed at the Göttingen Genomics Laboratory (G2L, Göttingen University, Germany) *via* a hybrid approach using the Genome Analyzer (GA) II and the MiSeq (Illumina). The shotgun library was prepared according to the manufacturers' protocol. This involved the use of the Nextera library preparation. The library was sequenced on the Genome Analyzer IIx using the TruSeq SBS Kit V5-GA and on the Miseq instrument using MiSeq reagent kit version 3 as recommended by the manufacturer (Illumina, San Diego, CA, USA). Sequencing resulted in 8,364,879 reads (2 × 112 bp) sequenced on the GA IIx and 11,075,962 reads (2 × 301 bp) on the MiSeq. The paired-end Illumina sequence reads were pre-processed using Trimmomatic with quality filter Phred33 (Bolger et al., 2014) resulting in trimmed sequence reads with a mean length of 92.7 and 201 bp, respectively.

*De novo* assembly of the total of 8,184,130 trimmed paired-end GA II and 10,406,346 MiSeq reads was achieved using SPAdes 3.7.0. (This version includes the metaSPAdes metagenomic pipeline, Bankevich et al., 2012). The assembly were inspected and quality checked using Qualimap (García-Alcalde et al., 2012).

Automated gene prediction and annotation was conducted with PROKKA (Seemann, 2014). Phylogenetic assignment of annotated contigs was achieved *via* Blast comparison with the NCBI database using Blobology (https://github.com/blaxterlab/blobology). These analyses were performed using the default settings of the various programs. This Whole Genome Shotgun project has been deposited at DDBJ/ENA/GenBank under the accession MLJW00000000. The version described in this paper is version MLJW01000000. The unassembled nucleotide sequence reads are available at the Sequence Read Archive of the NCBI *via* accession numbers SRR5040535 (GAII sequence reads) and SRR5040536 (MiSeq sequence reads).

Transmembrane helices in protein sequences were identified by TMHMM 2.0 using the default settings (Möller et al., 2001, http://www.cbs.dtu.dk/services/TMHMM/ which was accessed on 19 September 2016).

## Results and discussion

Attempts to enrich microaerophilic strains from AMD collected at the inflow into the treatment plant Tzschelln were based on the previous finding using culture-independent approaches that *Gallionella*-like strains were more abundant in the near-anoxic inflow AMD than in samples collected from the oxidation basin of the treatment plant Tzschelln (Gelhaar, 2012). These attempts resulted in various cultures of which enrichment culture ADE-12-1 (Figure 1A) was chosen for further metagenomic analysis. An aliquot of culture ADE-12-1 was transferred into an oxygen-ferrous iron-gradient in a 50-mL bottle (Figure 1B) in order to obtain sufficient biomass for subsequent isolation of total genomic DNA. Extraction of the total nucleic acid fraction of this 50-mL culture after 35 days of incubation provided 1.2 μg of DNA.

### Metagenome assembly of *Sideroxydans* related contigs

Illumina-based sequence analysis of the metagenomic DNA extracted from enrichment culture ADE-12-1 provided 20,563,010 GAII and 10,406,346 MiSeq sequence reads which were assembled into 9456 contigs totalling 56.8 Mb of metagenomic sequence information. Although, only 762 of these contigs were larger than 10 kb, these still represent approximately two thirds (38 Mb) of the total sequence information. Therefore, and due to the fact that the contigs < 10 kb encode only up to 10 ORFs further detailed analyses focused on contigs > 10 kb (Supplementary Table 1A). Key information on the metagenomic sequence data obtained for enrichment culture ADE-12-1 are summarized in Table 1.

**Table 1:** **Metagenomic sequence information obtained for the microaerophilic enrichment culture ADE-12-1**.

**General information on the metagenomic sequence data**	**Total**		***Sideroxydans***	
	**Value or range**	**Average**	**Value or range**	**Average**
Total sequence length (Mb)	56.8	–	10.1	–
Contigs	9456	–	92	–
Contigs > 10 kb	762^a^	–	64^b^	–
Total sequence length (Mb)^c^	38.1	–	9.8	–
Contig size (kb)^c^	10–901.1	49.9 kb	15.3–901.1	155
Coverage (fold)^c^	5.2–280.3	68.7^d^	73.9–222	150.2^d^
GC content (%)^c^	73.6–34.8	58.9^d^	51–61%	58.6%^d^
ORFs^c^	35,340	–	9673	–
ORFs per contig^c^	10–891	46.5	12–891	151.1

a *24 of these contigs could not be assigned to specific taxa*.

b *Contig GALL_all_contig000366 was removed from the dataset due to low coverage (8-fold); it also had the highest average GC content (61.5%)*.

c *Of those > 10 kb*.

d *The average was calculated taking the contig length into consideration (i.e., values per base)*.

Blast-based sequence comparison of the individual contig sequences assigned 738 of the 762 contigs (Supplementary Table 1A) to phylogenetic groups (in general to genus or species level) that were largely also detected by sequence analysis of PCR-amplified 16S rRNA gene fragments (Supplementary Table 2, Table 2). Only 64 of these remaining 738 contigs (8.8%) seem to have derived from *Sideroxydans* or *Sideroxydans*-like strains (Table 1, Supplementary Table 1B). However, these 738 contigs comprise approximately a quarter of the metagenomic sequence information (9.8 Mb of the 38 Mb of total metagenomic sequence data encoded within contigs > 10 kb: Table 1, Supplementary Table 1B) since most of the largest contigs are among those. For example, the 13 largest contigs assigned to *Sideroxydans* total more than half (5.01 Mb) of the sequence information encoded by the 64 contigs > 10 kb. Therefore, and despite 12 contigs being < 50 kb, the 64 contigs averaged 155 kb in size and were sequenced with an approximately 150-fold coverage. In total, the 64 contigs encode 9673 ORFs (Table 1).

**Table 2:** **16S rRNA based analysis of the bacterial diversity within the microaerophilic enrichment culture ADE-12-1**.

	**16S rRNA library^a^**	**16S-tag Illumina^b^**
–Total number of clones or sequence reads	100	42,639
–Most abundant taxa within ADE-12-1 and *Gallionella*		
• *Telmatospirillum*	14 (14%)	19,680 (46.2%)
• *Opitutus*	13 (13%)	9261 (21.7%)
• *Sideroxydans* (total)^c^	70 (70%)	4133 (9.7%)
OTU_31^d^	–	4131 (99.95%)
OTU_1160^d^	–	1 (0.002%)
OTU_1869^d^	–	1 (0.002%)
• *Gallionellaceae*	70 (70%)	4178 (9.8%)
Percentage *Sideroxydans* of *Gallionellaceae*^c^	100%	98.92%
• *Gallionella*^c^	0 (0%)	0 (0%)

a *Number of clones screened by ARDRA with subsequent sequence analysis of representatives of each ARDRA group*.

b *Numbers represent 16S-tag sequence reads*.

c *Only those reads were considered that were assigned to genus level*.

d *Of total Sideroxydans reads*.

### Bacterial diversity within the AMD sample and within the microaerophilic enrichment culture ADE-12-1

The metagenomic dataset (Table 1) contains only 13 contigs that harbor a 16S rRNA gene or gene fragment, with four instances in which the 16S rRNA sequence was part of a large contig (> 100 kb). In the other cases the 16S rRNA sequences were individual sequence reads or within small contigs (up to ca. 32 kb, Supplementary Table 3). This is not surprising since ribosomal gene clusters often occur in multiple copies within genomes which, in turn, affects the assembly of the raw data. The genomes of the microaerophilic strains *S. lithotrophicus* ES-1 and *G. capsiferriformans* ES-2, are, for instance, known to harbor two (ES-1) or three (ES-2) ribosomal RNA operons (Emerson et al., 2013). Blast analysis of these 16S rRNA gene fragments from the metagenomic dataset confirmed the presence of eight genera (*Sideroxydans, Telmatospirillum, Cellulomonas, Sulfuritalea, Sediminibacterium, Thiomonas, Methylotenera, Opitutus*) from four phyla (*Proteobacteria, Actinobacteria, Bacteroidetes, Verrucomicrobia*). However, the 16S rRNA gene fragments of neither neutrophilic *Gallionella* nor acidophilic *Gallionella*-like strains (Heinzel et al., 2009a) were closest (BLAST) hits to any of the 16S rRNA sequences detected in the metagenomic data. One of the 16S rRNA gene fragments showed highest sequence similarity to *Sideroxydans lithotrophicus* strain ES-1 (Supplementary Table 3).

**Figure 2:**
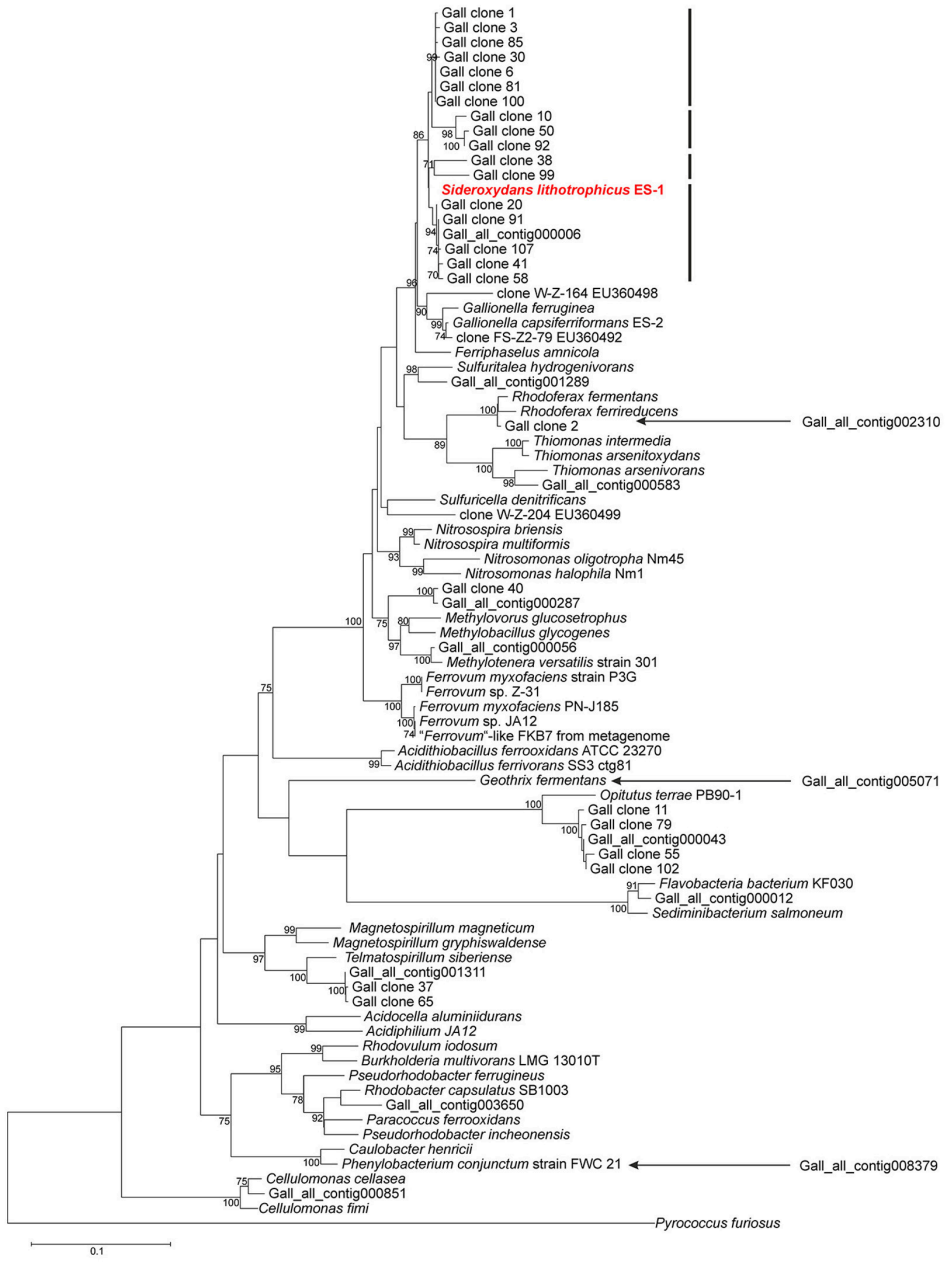
**Evolutionary relationship of strains detected within culture ADE-12-1 based on an alignment of 16S rRNA gene fragments**. Sequences were obtained from the metagenomic dataset (see Supplementary Table 3) and from the sequence analysis of the clone library of 16S rRNA gene fragments (see Table 2). The position of three 16S rRNA gene fragments (out of the total of 13) from the metagenomic dataset was determined separately due to the short overlapping region (ca. 205 bp) with the other sequences. This was achieved by adding them to an identical neighbor-joining tree using the parsimony option within ARB. Their positions within the tree are indicated by arrows. Calculation of phylogenetic trees was conducted within MEGA 6 (Tamura et al., 2013). The position of *S. lithotrophicus* ES-1 is highlighted in bold and red characters.The bars next to the position of *S. lithotrophicus* ES-1 and related strains indicate potential clusters. The evolutionary history was inferred using the Neighbor-Joining method in combination with the Jukes-Cantor model (Saitou and Nei, 1987) and bootstrap tests (1000 replicates; Felsenstein, 2004). Only bootstrap values > 70 % are shown next to the branches. There were a total of 683 positions in the final dataset. The 16S rRNA gene fragment of *Pyrococcus furiosus* was used as outgroup.

To obtain more information on the bacterial diversity within enrichment culture ADE-12-1, sequence analysis of PCR-amplified 16S ribosomal RNA gene fragments was used in a two-tier approach that aimed at providing both: robust assignment of sequence reads to specific taxa (i.e., Sanger sequence analysis of clones from a clone library of large gene fragments) and (near) complete coverage of the diversity (i.e., 16S-tag Illumina sequence analysis). Blast-based comparison of the nucleotide sequences obtained from the clone library of approx. 1300-bp 16S rRNA gene fragments with the entries in the SILVA database (Pruesse et al., 2007) confirmed that *Sideroxydans*-like strains were abundant in enrichment culture ADE-12-1 and that no close *Gallionella* strains seem to be present (Table 2). Although, the extent of their dominance was found to be lower, *Sideroxydans* strains still represent almost 10 % of the bacterial cells within enrichment culture ADE-12-1 when analyzed by 16S-tag Illumina sequencing (Table 2), an approach that avoids a potential bias caused by the preparation of a clone library (i.e., cloning). Taxa that, based on the 16S-tag Illumina sequencing approach, were found to dominate enrichment culture ADE-12-1 were *Telmatospirillum* (*Alphaproteobacteria*) and *Opitutus* (*Verrucomicrobia*) (Table 2). Again, *Gallionella*-derived sequences were not detected among the 42,639 Illumina sequence reads (Table 2). A phylogenetic analysis of the 16S rRNA sequences from the clone library and from the metagenomic data further confirmed the results and indicates the presence of at least three to four different *Sideroxydans* and *Sideroxydans*-like groups of strains (Figure 2).

In summary, the application of the gradient tube technique proved to be successful for the cultivation and enrichment of microaerophilic iron-oxidizing *Sideroxydans*-like strains from AMD. The absence of any *Gallionella* strains raises, however, the question as to the reasons for the observed discrepancy to the results previously reported for the same environment; that is, the abundance of *Sideroxydans* strains *versus* that of *Gallionella*-like strains (Heinzel et al., 2009a,b; Gelhaar, 2012). Clearly, the samples tested in previous studies (Heinzel et al., 2009a,b) and that used as inoculum for enrichment culture ADE-12-1 were collected at different times (2007 vs. 2014, respectively) and the observed results may indeed reflect a shift in the bacterial community within the AMD inflow to the treatment plant Tzschelln. However, the methodological approaches used by Heinzel et al. (2009a,b) for the analysis of the bacterial diversity (ARDRA analysis of clones from a library of 16S rRNA gene fragments: Heinzel et al., 2009a) and the quantification of *Gallionella*-like strains (terminal restriction fragment length polymorphism (TRFLP) analysis and real-time qPCR: Heinzel et al., 2009b) were also scrutinized in order to uncover potential causes for the different results. This examination revealed that neither the ARDRA nor the TRFLP approach distinguishes between *Gallionella* and *Sideroxydans* strains (results not shown). In contrast to this, the real-time qPCR approach withstands this scruntiny, in particular due to a mismatch between the 3′-end base of reverse primer 384r (Heinzel et al., 2009b) and its corresponding binding site within *Sideroxydans* strains (results not shown). The discrepancy between the results obtained by Heinzel et al. (2009b) from real-time qPCR and TRFLP analyses may, therefore, be explained by the possible detection of *Sideroxydans* strains in the samples from the treatment plant Tzschelln by one (TRFLP) but not the other (real-time qPCR) methodological approach.

Nevertheless, the culture conditions applied and the observation of rust-like precipitates, which most likely represent ferric iron hydroxy-like compounds, and the close 16S rRNA sequence similarity to that of *S. lithotrophicus* strain ES-1 indicate that microaerophilic iron-oxidizing microorganisms were enriched that can withstand low pH conditions. Moreover, the metagenomic sequence data obtained for enrichment culture ADE-12-1 proved to be particularly relevant in defining adaptive strategies to pH homeostasis, since the family *Gallionellaceae* had previously only been known to consist of the microaerophilic and neutrophilic iron oxidizers *Gallionella* and *Sideroxydans*. That is, the availability of the complete genome of the neutrophilic *S. lithotrophicus* strain ES-1 (Emerson et al., 2013) now permits the detailed comparison of the metabolic capacity of neutrophilic and acidophilic members of the same genus and, thus, the detection of biochemical features that are present in acidophilic strains to support life under acidic conditions. Attempts were therefore undertaken to reconstruct the metabolic potential of these acidophilic strains from the metagenomic sequence data assigned to *Sideroxydans* strains.

### Metabolic reconstruction from *Sideroxydans* metagenomic contigs

Reconstruction of metabolic traits was based on the analysis of the automated annotation of the 9673 ORFs assigned to members of this genus (Table 1) and provided new insights into the life style of acidophilic representatives of the genus *Sideroxydans*. Overall, the analysis of this fraction of the metagenomic dataset focused on nutrient assimilation, energy production and strategies for pH homeostasis. The findings reported here indicate that acidophilic *Sideroxydans* strains have a wider repertoire of metabolic features available in this respect than found in the genome sequence of the neutrophilic *S. lithotrophicus* strain ES-1 (Emerson et al., 2013). However, this has to be seen in the context of the obvious imbalance of available genome data of neutrophilic strains (i.e., only that of strain ES-1) versus the metagenome comprised of presumably several strains. This has to be taken into account throughout the following discussions which directly compare metabolic features reconstructed from the metagenomic dataset with those of the neutrophilic strain ES-1. In this context, it should be noted that reference is made to acidophilic *Sideroxydans* strains independent of the fact that the genomic and metabolic features which are discussed may be present only in one strain or are shared by several strains. Similarly, it remains inconclusive whether all or only some of the metabolic features are present within a particular strain.

#### Nutrient assimilation and biomass production

As mentioned above, the metagenomic sequence data assigned to acidophilic *Sideroxydans* strains indicate a more extensive metabolic potential for nutrient assimilation than predicted for the neutrophilic strain ES-1 based on its genome sequence. An example for this is provided by the fact that strain ES-1 seems to be limited to inorganic phosphate as source of phosphorus while acidophilic *Sideroxydans* strains represented in the metagenomic dataset appear to have the potential to use phosphonates in addition to inorganic phosphate. Contig Gall_all_000077 contains a gene cluster comprising 14 genes (GALL_all_158100 - GALL_all_158230) which encode an ABC transporter for phosphonate uptake, a phosphonate lyase (C-P lyase, encoded by *phnJ*) together with all required accessory proteins (encoded by *phnGHIKLMNP*) (Supplementary Table 4A). In contrast to this, the genome of the neutrophilic strain ES-1 does not contain a homolog of this gene cluster, but only encodes three proteins with high sequence similarity to phosphonate uptake (Slit_1183) or to putative phosphonate utilization (Slit_1659, Slit_2972). In any case, psi-Blast searches with C-P lyases as query, including that from the metagenomic dataset, did not reveal any C-P lyase gene within the *Sideroxydans* ES-1 genome, thus indicating that, if strain ES-1 should be able to utilize phosphonates, it would be *via* a different route. Interestingly, the close localization of genes encoding a tRNA(-Met) (GALL_all_158020) and a transposase (GALL_all_158310) upstream and downstream of the phosphonate encoding gene cluster on contig000077, respectively, indicates that this gene cluster in the acidophilic *Sideroxydans* strain may have been acquired *via* horizontal gene transfer. Similarly, a transposase and an integrase are also located upstream and downstream of the phosphonate encoding gene cluster in the genome of *G. capsiferriformans* strain ES-2 which is highly similar to that on contig_000077. A possible explanation for this observation may be that the common ancestor of the *Gallionellaceae* has acquired the genetic ability to utilize phosphonates as a source of phosphorus which was later lost by the neutrophilic *S. lithotrophicus* strain ES-1. Following its uptake phosphate can then be stored in form of polyphosphate granules synthesized by the polyphosphate kinase (encoded by *ppk*) and liberated upon cellular phosphate depletion (*via* action of an exopolyphosphatase, encoded by *ppx*; Supplementary Table 4A).

Similar to the situation described for the utilization of phosphorus sources, the metagenomic sequence data assigned to *Sideroxydans* strains also indicate that the acidophilic strains seem to be more versatile regarding nitrogen assimilation than the neutrophile *S. lithotrophicus* ES-1, though—as mentioned above—the former may comprise several genetically different strains. The metagenomic dataset as well as the genome of strain ES-1 harbors genes that are predicted to code for the ability to assimilate ammonium, nitrate and nitrite and to fix elementary nitrogen *via* nitrogenase activity (Supplementary Table 4B). Genes related to nitrogen fixation are organized in several small gene clusters within each of the three contigs (contig000009, contig000013, contig000035) encoding a nitrogenase (Supplementary Table 4B), though all within close proximity to each other and to a Rnf complex encoding gene cluster. Expression of the nitrogenase may be regulated *via* reversible ADP-glycosylation of a specific arginine residue in the nitrogenase complex catalyzed by a dinitrogenase reductase glycohydrolase (DraG) and a dinitrogenase reductase ADP-ribosylation transferase (DraT). DraG and DraT are also encoded on contig000009 in close proximity to the nitrogenase. Such a mechanism has been described in detail for the alphaproteobacterium *Rhodospirillum rubrum* (Wang and Norén, 2006). Both the neutrophilic *Sideroxydans* strain ES-1 (Emerson et al., 2013) and the metagenomic data indicate that recycling (i.e., reduction) of ferredoxin, which is required by the dinitrogenase reductase as low potential reduction equivalents, is achieved *via* a Rnf membrane-integral protein complex. The energy for the uphill electron transport from NADH to ferredoxin, which is mediated by the Rnf complex, may be obtained through ion (H^+^ or Na^+^) influx across the membrane-bound Rnf complex (e.g., Biegel and Müller, 2010). However, the stoichiometric ratio of inflowing protons required for electron transfer from NADH to ferredoxin remains unclear since it depends on the prevailing membrane potential.

One of the acidophilic *Sideroxydans*-derived contigs (contig000112) also encodes a urease gene cluster (Supplementary Table 4B). The host cell of contig000112 therefore seems to be able to utilize urea as alternative nitrogen source, with urea being taken up *via* an ABC transporter (Gall_all_190370, Gall_all_190380, Gall_all_190390) encoded upstream of the urease gene cluster on the same contig (Supplementary Table 4B). In contrast to this, the genome of strain ES-1, which lacks a homolog of the urease gene cluster, harbors a four-gene cluster (Slit_0078 - Slit_0081) encoding an alternative urea utilization pathway which is not found among the *Sideroxydans*-assigned metagenomic contigs. This pathway involves urea carboxylase and allophanate hydrolase activity thought to be also involved in urea degradation (Hausinger, 2004; Kanamori et al., 2004, 2005). However, since this represents an ATP-dependent process and given the absence of obvious urea uptake mechanisms it may perhaps be more likely that this process is involved in a biosynthetic pathway (e.g., amino acid synthesis) in strain ES-1 rather than in urea utilization.

Assimilated nitrogen may subsequently be stored in form of cyanophycin *via* the action of two cyanophycin synthetases (CphA1, CphA2), both encoded next to each other (Supplementary Table 4B) and on three of the *Sideroxydans*-assigned contigs (contig000008, contig000020, contig000031). The fact that none of the metagenomic contigs encodes a cyanophycin degrading cyanophycinase appears less surprising given that cyanophycinase genes have so far not been detected in any of the available genomes of the *Betaproteobacteria* (Krehenbrink et al., 2002; Blast searches against the NCBI database were repeated on 23 August 2016 with the same result). Assuming that cyanophycin is synthesized in acidophilic *Sideroxydans* strains, this would point at the presence of an alternative pathway to liberate nitrogen and carbon stored in cyanophycin in order to meet cellular requirements (Krehenbrink et al., 2002). The presumption that cyanophycin also serves as carbon storage molecule is based on the fact that the genetic basis for the metabolic capacity to store carbon in form of, for instance, polyhydroxybutyrate granules has so far not been detected among the *Sideroxydans*-assigned metagenomic contigs. As is the case for urease activity, cyanophycin synthesis is not encoded by the neutrophilic strain ES-1, though the presence of cyanophycin synthetase genes (*cphA1, cphA2*) in the genome of *G. capsiferriformans* strain ES-2 may indicate that genome analysis of further neutrophilic *Sideroxydans* strains may reveal their presence also in neutrophilic strains. The ability to use cyanophycin as nitrogen storage is also encoded in the genomes of other genera comprised of both neutrophilic (e.g., *Desulfosporosinus orientis, D. youngiae, D. meridiei*: Pester et al., 2012) and acidophilic (e.g., *D. acididurans* M1: Petzsch et al., 2015, *D. acidiphilus*: Pester et al., 2012) species.

Based on the metagenomic datset it appears that sulfate, which is present in surplus in AMD, represents the sole source of sulfur taken up by a specific sulfate ABC transporter or a sulfate permease (Supplementary Table 4C). Sulfate is predicted to be subsequently assimilated *via* the adenosine phosphosulfate (APS) pathway. This involves sulfate activation by a sulfate adenylyltransferase to adenosine-phosphosulfate (APS) and subsequent reduction to sulfite and sulfide. The latter reaction is, similar to that predicted for the neutrophilic iron oxidizers *S. lithotrophicus* strain ES-1 and *G. capsiferriformans* ES-2, catalyzed by a ferredoxin-dependent sulfite reductase rather than a NADH-dependent sulfite reductase (Supplementary Table 4C).

Similar to the neutrophilic strain ES-1 carbon acquisition seems to be achieved *via* the Calvin-Benson-Bassham cycle with form I and form II of the ribulose-1,5-bisphosphate carboxylase/oxygenase (RuBisCO). Carbon dioxide fixation also relates to the fact that culture ADE-12-1 had been enriched under autotrophic conditions. RuBisCO form I is encoded on three contigs (contig000009, contig_000020, contig_000030: Supplementary Table 4D). Both small (CbbS) and large (CbbL) subunit of RuBisCO are clustered together in each of these cases. Addition of the CbbL amino acid sequences to the corresponding alignment produced by Badger and Bek (2008) and subsequent phylogenetic analysis indicates that all copies belong to RuBisCO form IAq (Supplementary Figure 1). Moreover, each of these three RuBisCO gene clusters also harbor genes for the RuBisCO-activating enzymes CbbQ and CbbO downstream of *cbbL/S*, but lacks any carboxysome encoding genes which is, again, characteristic for form 1Aq (Badger and Bek, 2008). RuBisCO form II (CbbM) is also encoded on three metagenomic contigs (contig000006, contig000023, contig000025) assigned to acidophilic *Sideroxydans* strains. These are within a cluster comprised of seven genes including the RuBisCO-activating proteins CbbQ and CbbO and a carbonic anhydrase, though, as mentioned above, no carboxysome encoding genes were detected in any of the *Sideroxydans*-assigned metagenomic contigs. The presence of both forms of RuBisCO is likely to provide higher tolerance to fluctuating O_2_ levels (Badger and Bek, 2008; Emerson et al., 2013) and even enable high CO_2_ fixation rates under anaerobic growth conditions. For example, in *A. ferrooxidans* ATCC 23270 (Appia-Ayme et al., 2006) and *T. denitrificans* ATCC 25259 (Beller et al., 2006) the two isoforms were found to be differentially expressed depending on the oxygen conditions and the electron donor (ferrous iron or reduced sulfur compounds) suggesting that RuBisCO form II is only produced under anaerobic conditions. However, the lack of completely reconstructed genomes from the metagenomic dataset means again that the question regarding the presence of RuBisCO form I and II genes in the same acidophilic *Sideroxydans* strain remains unresolved.

The product of carbon fixation *via* the CBB cycle is 3-phosphoglycerate which is likely to be further metabolized in the pathways of the central carbon metabolism in order to generate precursors of bacterial biomass polymers. For example, although not found within an individual metagenomic contig, the genetic basis for all relevant activities required for a functional tricarboxylic acid (TCA) cycle are present in the *Sideroxydans*-assigned metagenomic contigs. Again, the lack of completely reconstructed genomes from the metagenome does not allow to unambigously determine whether all of the acidophilic *Sideroxydans* strains within enrichment culture ADE-12-1 encode the complete rather than the incomplete TCA cycle, which had been thought to be typical for obligate chemolithoautotrophic prokaryotes (Wood et al., 2004). This “horseshoe” type TCA cycle (Wood et al., 2004) lacks the enzyme 2-oxoglutarate dehydrogenase. However, a complete TCA cycle is also present in the neutrophilic *Sideroxydans* strain ES-1 and has recently been detected in strains belonging to the proposed genus “*Ferrovum*” (Moya-Beltrán et al., 2014; Ullrich et al., 2016a,b). The two subunits (E1, E2) that encode the 2-oxoglutarate dehydrogenase (E1) and the dihydrolipoamide succinyltransferase (E2) activity, are located next to each other. The third subunit (E3) encoding the dihydrolipoamide dehydrogenase activity of the 2-oxoglutarate dehydrogenase enzyme complex is known to be often shared with the E1-E2 subunit sets of pyruvate dehydrogenase and of branched-chain α-keto acid dehydrogenases (Berg and de Kok, 1997; McCartney et al., 1998). Therefore, it appears little as surprise that subunit E3 does not cluster with the first two components of the 2-oxoglutarate dehydrogenase enzyme complex.

The presence of a complete TCA cycle has generally been regarded as a sign of a heterotrophic life style and, therefore, raises the question as to the possibility that iron-oxidizing *Betaproteobacteria*, so far thought to be obligate autotrophs, might be able to also assimilate and utilize organic compounds for growth. However, based on their genome sequences the *Proteobacteria Nitrosomonas europea* and *Rhodobacter capsulatus* seem to encode 2-oxoglutarate dehydrogenase, but are apparently not able to achieve heterotrophic growth (Wood et al., 2004). Thus, although, as mentioned above, both neutrophilic and acidophilic *Sideroxydans* strains seem to encode the complete TCA cycle, this may not confer heterotrophic growth (see Wood et al., 2004 for a discussion on possible reasons).

#### Energy metabolism

It is now widely accepted that iron oxidation pathways among ferrous iron-oxidizing bacteria are varied (e.g., Bonnefoy and Holmes, 2012; Ullrich et al., 2016a). However, it appears that the acidophilic *Sideroxydans* strains use the same pathway for energy production thought to be present in the neutrophilic strain ES-1 (Emerson et al., 2013). Automated annotation of the metagenomic sequence data did not indicate the presence of any genes related to iron oxidation. However, using psi-Blastp searches with those gene products as query that were found to be likely candidates for iron oxidation in *S. lithotrophicus* ES-1, revealed two metagenomic contigs (contig_000001, contig_000139) which encode gene clusters consisting of four genes each (Gall_all_14490 - Gall_all_14520 and Gall_all_209710 - Gall_all_209740, respectively) with high sequence similarity to the corresponding gene cluster in strain ES-1 (Table 3, Supplementary Table 4E). These genes represent homologs to the genes encoding MtrA/B in *Shewanella oneidensis* MR-1 and PioA/B genes in *Rhodopseudomonas palustris* TIE-1 which are involved in iron reduction and photoferrotrophy, respectively (Liu et al., 2012; Emerson et al., 2013). Moreover, MtoA, the gene product of the *mtrA* homolog from *S. lithotrophicus* ES-1, has experimentally been verified to be a decaheme cytochrome with Fe(II) oxidizing activity in *in vitro* assays (Liu et al., 2012). The model formulated based on these findings (Liu et al., 2012; Emerson et al., 2013) suggests that MtoA (Slit_2497/ Gall_all_14490) together with MtoB (Slit_2496/ Gall_all_14500) and the CymA (Slit_2495/ Gall_all_14510) homolog represent the Fe-oxidizing complex (in *S. lithotrophicus* ES-1/acidophilic *Sideroxydans* strains, Emerson et al., 2013). Additionally, both gene clusters on the two metagenomic contigs (contig_000001, contig_000139) also encode the cytochrome c-type protein upstream of *mtoA*, but lack a homolog of the hypothetical protein found to be present downstream of *cymA* in *S. lithotrophicus* ES-1 (Emerson et al., 2013).

**Table 3:** **Result from psi-Blastp comparison of gene products encoded by a gene cluster in the genome of ***S. lithotrophicus*** ES-1 that is thought to be involved in iron oxidation, with gene products assigned to acidophilic ***Sideroxydans*** strains**.

	**Contig**		**CytC**	**MtoA**	**MtoB**	**CymA**	
*S. lithotrophicus* ES-1	^a^		Slit_2498	Slit_2497	Slit_2496	Slit_2495	Slit_2494
ADE-12-1	000003		–	Gall_all_14490	Gall_all_14500	Gall_all_14510	Gall_all_14520
		score^b^		383/4e^−136^	671/0.0	517/0.0	182/e^−59^
		annotated as^c^		denitrification system component NirT	hypothetical protein	cytochrome c nitrite reductase pentaheme subunit	cytochrome c2
	000139		–	Gall_all_209740	Gall_all_209730	Gall_all_209720	Gall_all_209710
		score^b^		384/e^−136^	653/0.0	521/0.0	190/e^−62^
		annotated as^c^		denitrification system component NirT	hypothetical protein	cytochrome c nitrite reductase pentaheme subunit	cytochrome c551 subunit

a *closed genome*.

b *shown is score/E value from psi-Blastp with five iterations*.

c *result from automated annotation*.

The metagenomic contigs assigned to *Sideroxydans* strains further encode, in addition to cytochrome *bc*, the alternative complex (AC) III (ACIII, Supplementary Table 4E) that is also present in *S. lithotrophicus* ES-1 (Emerson et al., 2013). However, the precise pathway of the electrons from the outer membrane across the periplasm to these proteins at the cytoplasmic membrane remains unresolved within all *Gallionellaceae*. That is, homologs to neither rusticyanin nor to the soluble cytochromes Cyc1 and CycA-1 of *A. ferrooxidans* ATCC23270 were detected on the metagenomic contigs assigned to acidophilic *Sideroxydans* strains. A similar scenario has been reported previously following a detailed analysis of the genomes of *S. lithotrophicus* ES-1 and *G. capsiferriformans* ES-2 (Emerson et al., 2013). Additional analyses using the amino acid sequence of the c-type cytochrome of “*Ferrovum*” sp. strain JA12 (Ferro_ 02680), that was found to have sequence similarity to Cyc1 (AFE_3152) of *A. ferrooxidans* ATCC23270 (Ullrich et al., 2016a), as template for a psi-Blastp search against the metagenomic *Sideroxydans* contigs revealed a potential homolog (Gall_all_02140, Gall_all_16150, Gall_all_65820) on three contigs (contig000001, contig000003, contig000019, respectively). These proteins, thought to be *c4*-type cytochromes, seem to be soluble based on the prediction obtained from the structural analysis using the TMHMM tool (Möller et al., 2001, Supplementary Figures 2A–C). Moreover, a further *c*-type cytochrome (Gall_all_02130, Gall_all_16160, Gall_all_65830) was found to be encoded next to each of these soluble cytochromes, though no homologs to the CycA-1-like proteins in “*Ferrovum*” sp. strain JA12 were detected in the *Sideroxydans*-assigned contigs. TMHMM-based predictions (Möller et al., 2001) indicate that these further *c*-type cytochromes are soluble, but anchored to a membrane at their N-terminal end (Supplementary Figures 2E–G). Should these two cytochromes (i.e., the soluble *c4*-type and the anchored *c*-type cytochrome) indeed accomplish the electron transport from MtoA across the periplasm to the cytochrome *bc* complex at the cytoplasmic membrane, then the following scenario might represent the electron transfer path: the *c*-type cytochrome is anchored at the outer membrane where it receives the electrons from the extracellular MtoA. The soluble Cyc1-like *c4*-type cytochrome would then shuttle the electrons to the cytoplasmic membrane where it transfers them directly or *via* cytochrome *bc* to the ubiquinol pool. Finally, the electrons are subsequently directed either upstream for the reduction of oxidized NAD(P)^+^ or downstream for the formation of a proton motive force.

As for the latter, five of the *Sideroxydans*-assigned contigs (contigs000003, contig000006, contig000019, contig000021, contig000215) encode F_0_F_1_-type ATPases, while one contig (contig000021) encodes a V-type ATPase (Supplementary Table 4E). In each of these cases, the genes encoding the various subunits of the ATP synthetases are organized within a gene cluster.

**Figure 3:**
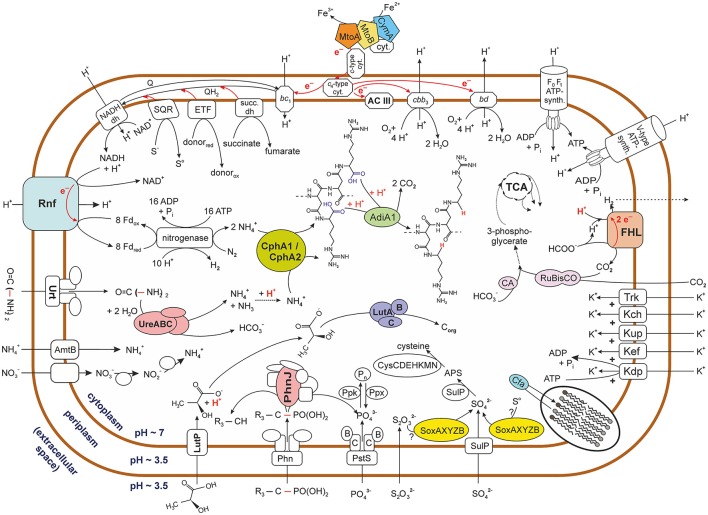
**Graphical representation of potential metabolic activities of acidophilic ***Sideroxydans*** strains**. The figure represents the pool of metabolic pathways that were reconstructed from the metagenomic sequence data of contigs assigned to strains of the genus *Sideroxydans*. It therefore does not express the co-occurrence of various pathways within the same strain. Biochemical reactions do not reflect real stoichiometry with the exception of cases were it was judged to be of relevance. Question marks indicate that it is unknown which sulfur compound is oxidized *via* the Sox enzyme system. Based on its pKa (3.86) lactate is depicted in its acid form outside the cell, though the narrow difference to the external pH (3.5) means that only a small fraction of molecules will be protonated. Please also note in this context that, no lactate was added to the culture medium. Dotted arrows indicate that no details are provided on the multiple enzymes involved in the pathway while dashed lines indicate the potential path of a volatile compound (i.e., H_2_). For reasons of clarity no details are provided on the tricarboxylic acid (TCA) cycle. Arrows deviating from the the TCA cycle indicate its relevance as a central carbon metabolic pathway, though this line of metabolic function was not further investigated in this study. Carbon dioxide appears to be reduced to 3-phosphoglycerate *via* the Calvin-Benson-Bassham cycle indicated by its key enzyme ribulose-1,5-bisphosphate carboxylase/oxygenase (RuBisCO). Names for the nitrate transporter and nitrate and nitrite reductases are not indicated due to contradicting nomenclature in the databases. FHL, formate hydrogenlyase; AC III, alternative complex III; ETF, electron transfer flavoprotein oxidoreductase; SDH, succinate dehydrogenase; SQR, sulfide quinone oxidoreductase; CA, carbonic anhydrase; NADH dh, NADH dehydrogenase; Phn, phosphonate uptake system. Enzymes involved in metabolic pathways are abbreviated by their acronyms. For details see Supplementary Table 4.

Apart from ferrous iron oxidation, the *Sideroxydans* metagenomic dataset also harbors three contigs (contig000005, contig000007, contig000011) with a cluster comprised of 12 genes that seem to encode proteins involved in dissimilatory sulfur oxidation (Supplementary Table 4E), including *dsr* genes. These Dsr enzymes have been shown to function in reversible (i.e., oxidative) manner to those found in sulfate reducing bacteria (Friedrich et al., 2005; Ghosh and Dam, 2009; Watanabe et al., 2014). This together with a contig (contig000067) encoding a gene cluster that harbors SoxXYZAB (Figure 3, Supplementary Table 4E) indicates that oxidation of reduced sulfur compounds may provide an additional path to gain energy for metabolic activities. Additional and more detailed analyses are likely to reveal further genes involved in the dissimilatory oxidation of sulfur compounds.

The *Sideroxydans*-assigned metagenomic contigs also encode enzymes for other redox reactions connected to the quinol pool. Among those are a predicted succinate dehydrogenase, an electron transfer flavoprotein (ETF) and a predicted flavoprotein dehydrogenase (ETF ubiquinone oxidoreductase) (Figure 3, Supplementary Table 4E).

Additionally, two (contig000035, contig000112) of the metagenomic contigs assigned to *Sideroxydans* also harbor a cluster comprised of five genes (*pdhR, lutABC, lutP*) that have sequence similarity to a GntR-type transcriptional repressor (PdhR, predicted to be involved in pyruvate metabolism), the lactate utilization proteins A, B and C and a lactate permease, respectively (Supplementary Table 4E). The lactate utilization complex ABC has been shown to be essential for growth of *Bacillus subtilis* on lactate as sole carbon source, while a *B. subtilis* mutant lacking the lactate permease only grew at a very slow rate (Chai et al., 2009). A low-level uptake of L-lactate *via* Na^+^ or K^+^ symporters has been discussed as possible reason for growth despite the absence of a functional lactate permease (Chai et al., 2009). Such a low-level lactate uptake may also be relevant for *S. lithotrophicus* strain ES-1 since its genome likewise encodes the lactate utilization complex LutABC and the GntR-type transcriptional repressor, but lacks a lactate permease. However, in contrast to *B. subtilis* neither the genome of *S. lithotrophicus* strain ES-1 nor the metagenomic contigs assigned to acidophilic *Sideroxydans* strains seem to encode a lactate dehydrogenase required for channeling the organic carbon into biosynthetic pathways or to make it available for a fermentative metabolism during anaerobic growth. This together with the fact that each of the three components of the lactate utilization complex in strain ES-1 or on the metagenomic contigs was inferred to contain an iron-sulfur cluster indicates that a cytochrome electron transfer is associated to lactate oxidation (i.e., respiration) rather than a biosynthetic pathway (i.e., heterotrophic growth) or NAD^+^ recycling (Chai et al., 2009).

However, apart from the question regarding availability of lactate in typical AMD environments, growth of acidophilic strains under acidic conditions on organic acids also appears unusual as this is likely to damage the proton gradient across the cytoplasmic membrane and, hence, the proton motive force required for ATP synthesis. Organic acids like lactate are protonated under acidic pH conditions (e.g., in AMD), but release a proton upon entering the cytoplasm which is thought to have circumneutral pH and, thus, result in an import of protons (Alexander et al., 1987; Kishimoto et al., 1990; Ciaramella et al., 2005). A solution to this issue may be provided by a scenario in which the lactate utilizing enzyme complex is located at the periplasmic site of the inner membrane, while the lactate permease permits lactate entry into the periplasm.

The homologous gene cluster in *B. subtilis* also encodes a GntR-type transcriptional repressor (LutR), though this has only low sequence similarity to the GntR-type transcriptional repressor (PdhR) in contig000035 and contig000112. Lactate utilization in *B. subtilis* is under the dual control of LutR and the master regulator for biofilm formation SinR, and is induced in response to L-lactate availability (Chai et al., 2009). No SinR homolog was found in any of the *Sideroxydans*-assigned metagenomic contigs, indicating that the lactate utilization complex in the host cells of contig000035 and contig000112 transfers metabolic ability for energy production *via* lactate-utilization, but does not play a role in biofilm formation.

*Sideroxydans* strains as well as other microaerophilic species may encounter anoxic periods during which respiration with oxygen as terminal electron acceptor is no longer possible. Based on the metagenomic dataset acidophilic *Sideroxydans* strains seem to be able to produce energy during anaerobic growth *via* at least one respirative path. The presence of a gene cluster (GALL_all_03470 - GALL_all_03520: Supplementary Table 4E) encoding the formate hydrogenlyase (FHL) complex indicates that acidophilic *Sideroxydans* strains may utilize formate oxidation during anoxic episodes to subsequently channel electrons *via* NADH ubiquinone oxidoreductases in the respiratory system to protons. The fact that the FHL gene cluster also encodes the subunits of a group 4 hydrogenase (Vignais et al., 2001) further corroborates the notion that formate oxidation coupled to the reduction of protons plays a role in energy conservation under oxygen-limiting conditions. Since the overall driving force for formate oxidation is rather low (18 mV at pH 7 and equal partial gas pressure of CO_2_ and H_2_), the acidic environment of acidophilic *Sideroxydans* strains means that it increases by 30 mV for each drop in pH unit (McDowall et al., 2014). Should this process still not yield any energy, then it may at least represent another means of reducing the intracellular proton load by proton reduction to volatile H_2_. However, more detailed analyses of all hydrogenases present in the metagenomic dataset might reveal further insights into electron transfer pathways and whether these have energy conserving potential. While the genome of *G. capsiferriformans* ES-2 encodes a pyruvate formate lyase (Galf_0602) for the conversion of pyruvate to acetyl-CoA and formate under anoxic conditions, a corresponding gene was neither detected in any of the *Sideroxydans*-assigned metagenomic contigs nor in the genome of the neutrophilic *Sideroxydans* strain ES-1. It remains speculative whether the pyruvate formate lyase is encoded on genomic fragments not covered by this metagenomic sequencing approach.

#### Strategies to adapt to life at low pH

Analyses of the metagenomic sequence information of *Sideroxydans*-derived contigs indicate a repertoire of strategies to deal with the acidic environment. Several of those mechanisms are also encoded on the genome of the neutrophilic *S. lithotrophicus* strain ES-1. An example of those is the cyclopropane-fatty-acyl-phospholipid synthase (Supplementary Table 4F) which enables the cell to produce and subsequently incorporate cyclopropane fatty acids into its cell membrane, thus protecting against proton influx (Grogan and Cronan, 1997; Chang and Cronan, 1999; Mangold et al., 2013). In addition to its potential to produce energy, the above mentioned formate hydrogenlyase activity also consumes one proton per molecule formate and may, thus, provide a means of buffering against high intracellular proton concentrations. Furthermore, creating a reversed, that is inside positive, membrane potential resembles a well-known strategy in pH homeostasis (Baker-Austin and Dopson, 2007). Both neutrophilic strain ES-1 and the *Sideroxydans*-assigned metagenomic contigs encode four uptake systems for potassium (the low affinity potassium transport system protein Kup, the voltage-gated potassium channel Kch, the Trk potassium uptake system and the Kef-type K^+^ transport system, see Supplementary Table 4F) which is considered an effective agent in achieving reversal of the membrane potential.

However, detailed analysis of the metagenomic data also revealed the presence of genes encoding three putative cellular functions that seem to be unique to the acidophilic representatives of *Sideroxydans*. Firstly, the aforementioned potassium uptake systems achieve potassium transfer into the cytoplasm only at high extracellular potassium concentrations (e.g., Damnjanovic et al., 2013). In contrast to this, three metagenomic contigs (contigs000001, contig000049, contig000055) encode an additional Kdp-type K^+^ uptake ATPase. This high-affinity potassium uptake system confers K^+^ uptake into the bacterial cytoplasm even under low environmental K^+^ concentrations (i.e., [K^+^]out < 100 μM), and, thus, maintains the cytoplasmic concentrations needed for, among other functions, pH homeostasis (Laimins et al., 1978; Epstein, 1985). In this ion pump, coupling of ATP hydrolysis to ion transport leads to a high-affinity uptake of potassium, though only at moderate transport rates and at the cost of ATP hydrolysis (Rhoads et al., 1976). Such a high-affinity potassium uptake system was not detected in the genome of *S. lithotrophicus* strain ES-1.

Secondly, and again in contrast to the genome of the neutrophilic strain ES-1, a gene cluster of 13 genes within contig000112 (Supplementary Table 4B) encodes both a urease and its accessory proteins as well as an ABC transporter for urea. Apart from providing the ability to utilize an alternative nitrogen source, urease activity has been known for some time to enable human pathogens, such as *Helicobacter pylori* (Eaton et al., 1991) and *Yersinia enterocolitica* and *Morganella morganii* (Young et al., 1996), to buffer against a high intracellular proton load. This buffering capacity is achieved through the urease catalyzed hydrolysis of urea which results in bicarbonate and ammonia (Mobley and Hausinger, 1989) and has recently also been proposed for “*Ferrovum*” group 2 strains JA12 and PN-J185 (Ullrich et al., 2016a,b). Since the gene cluster also encodes an ABC transporter for urea, it should further be mentioned that degradative processes of fossil organic matter within lignite similar to those processes reported for bioweathering of organics within copper shale (Matlakowska and Skłodowska, 2011; Matlakowska et al., 2012) may provide (traces of) urea in AMD.

The third feature which—based on the currently available genome data—seems to be unique to acidophilic *Sideroxydans* strains is related to the presence of two genes encoding the cyanophycin synthetases CphA1 and CphA2. Cyanophycin synthetase activity results in the non-ribosomal synthesis of the branched polypeptide cyanophycin which consists of aspartic acid in the backbone and an approximately equimolar amount of arginine in the side chain (Simon and Weathers, 1976). These genes are clustered together and found on three contigs (Supplementary Table 4B). Although, the presence of cyanophycin has been known for some time to occur in bacteria other than cyanobacteria (Krehenbrink et al., 2002), its potential role in pH homeostasis has not yet been discussed. The hypothesis put forward here is built on the facts that decarboxylation of amino acids is a well-known strategy in pH homeostasis (Baker-Austin and Dopson, 2007) and that the pKa values of the alpha-carboxy groups of the arginine residues is much lower (2.17, Campbell and Farrell, 2009) than the pH of the cytoplasms which is thought to be circumneutral. Decarboxylation of the deprotonated alpha-carboxy groups of arginine side chains in cyanophycin would then buffer against acidity. The presence of a biodegradative arginine decarboxylase (encoded by *adiA1* on contig001871) provides further support for this hypothesis. A contig (contig09308) encoding a cyanophycin synthetase and three contigs (contig01791, contig01794, contig04721) encoding degradative arginine decarboxylases were also detected in the metagenome constructed from a planktonic cell fraction of samples collected at an acid mine drainage (pH 2.5–2.7) stream biofilm situated 250 m below ground in the low-temperature (6–10°C) Kristineberg mine in northern Sweden (Liljeqvist et al., 2015). Moreover, since nitrogen does, in contrast to phosphate, not seem to be limiting in AMD, acidophiles may be able to accumulate large reservoirs of cyanophycin which may, in turn, not only represent a massive nitrogen and carbon reserve, but also an extensive buffer capacity against protons. Although, cyanophycin is widely known to occur in granular form in cyanobacteria, more recent research has shown that alterations to the side chains, such as incorporation of 5% lysine, results in a form soluble in an aqueous milieu (Frommeyer and Steinbüchel, 2013). Soluble cyanophycin is likely to be particularly accessible for enzymatic decarboxylation and, hence, might enable an almost instant response to high proton influx.

## Concluding remarks

Although, no pure culture or one that, similar to previous genomic studies on “*Ferrovum*” (Ullrich et al., 2016a,b), is composed of only the target microorganisms and a single contaminant, the availability of approx. 10 Mb of metagenomic sequence information derived from *Sideroxydans* strains provides a basis for the reconstruction of metabolic features present in acidophilic representatives of this genus. However, it must be reiterated in this context that the conclusions drawn from the analysis of the metagenomic data is limited by the lack of completely assembled genomes. Consequently, it remains unresolved which of these features are encoded by the same genome and to what extent the metagenomic assembly reflects genomic and, hence, metabolic variation of individual strains represented by the sequence data. While the latter might indicate niche partitioning by various ecotypes within the genus *Sideroxydans*, the presence of redundancy within a strain (e.g., different metabolic pathways for energy production) seems to be a useful means to survive in a changing environment. Overall, the results from this analysis demonstrate genetic differences to the neutrophilic *Sideroxydans* strain ES-1 that relate to interactions of acidophilic *Sideroxydans* strains with the prevailing environmental conditions of their specific habitats. The findings from this study therefore suggest an evolutionary process driven by the adaptation to distinct environmental niches, which presumably results in ecological speciation. Whether acidophily or neutrophily is the more evolved life style is, however, still open to discussion.

Clearly, this report has not covered all information on acidophilic *Sideroxydans* strains available within the metagenomic sequence data, most likely not even all of that relevant for nutrient utilization, energy production and pH homeostasis. Moreover, life in a typical AMD environment also means increased cellular damage caused by, for instance, higher rates of oxygen radical formation due to the high concentrations of redox active metals (in particular iron). Therefore, the genomes of acidophiles typically encode numerous mechanisms for the detoxification of reactive oxygen species (ROS, see Ferrer et al., 2016) and the repair of damaged biomolecules (e.g., genomic DNA itself). However, a detailed analysis of these aspects was seen as beyond the remit of this article, though an initial screening of the metagenomic sequence information indicates that the acidophilic *Sideroxydans* strains also encode the genetic potential for ROS detoxification and DNA repair.

Finally, sequence information on other members present in enrichment culture ADE-12-1 has also not yet been challenged and their role, therefore, remains likewise unresolved. Detailed analysis of—at least—the other two abundant groups (*Opitutus, Telmatospirillum*) may elucidate some of the reasons for their enrichment under the microaerophilic conditions within the gradient tubes. Preliminary analysis of the metagenomic contigs assigned to *Opitutus* and *Telmatospirillum* strains indicate that similar life styles to those known for characterized representative strains of these genera are also encoded by the strains within enrichment culture ADE-12-1 (e.g., methylotrophy: Chin et al., 2001; Sizova et al., 2007). However, more detailed analyses of the metagenomic data along these lines of investigation were again beyond the scope of this article.

## Author contributions

The study was proposed and designed by MM. AS produced enrichment culture ADE-12-1 and isolated the total metagenomic DNA. AP and RD planned metagenome sequencing. AP conducted sequence analyses including assembly and automated annotation of the genome reads. MV and MM analyzed the microbial diversity within enrichment culture ADE-12-1. MM analyzed the annotated metagenome data and wrote the manuscript. MS, RD, and AP contributed to the final manuscript by critical revision. All authors read and approved the manuscript and declare that the research was conducted in the absence of any commercial or financial relationships that could be construed as a potential conflict of interest.

### Conflict of interest statement

The authors declare that the research was conducted in the absence of any commercial or financial relationships that could be construed as a potential conflict of interest.
