# Supplementary figures and images for: Reconstruction of the Metabolic Potential of Acidophilic Sideroxydans Strains from the Metagenome of an Microaerophilic Enrichment Culture of Acidophilic Iron-Oxidizing Bacteria from a Pilot Plant for the Treatment of Acid Mine Drainage Reveals Metabolic Versatility and Adaptation to Life at Low pH
Source: Front Microbiol. 2016 Dec 22;7:2082. doi: 10.3389/fmicb.2016.02082 (PMC5178258; doi:10.3389/fmicb.2016.02082)

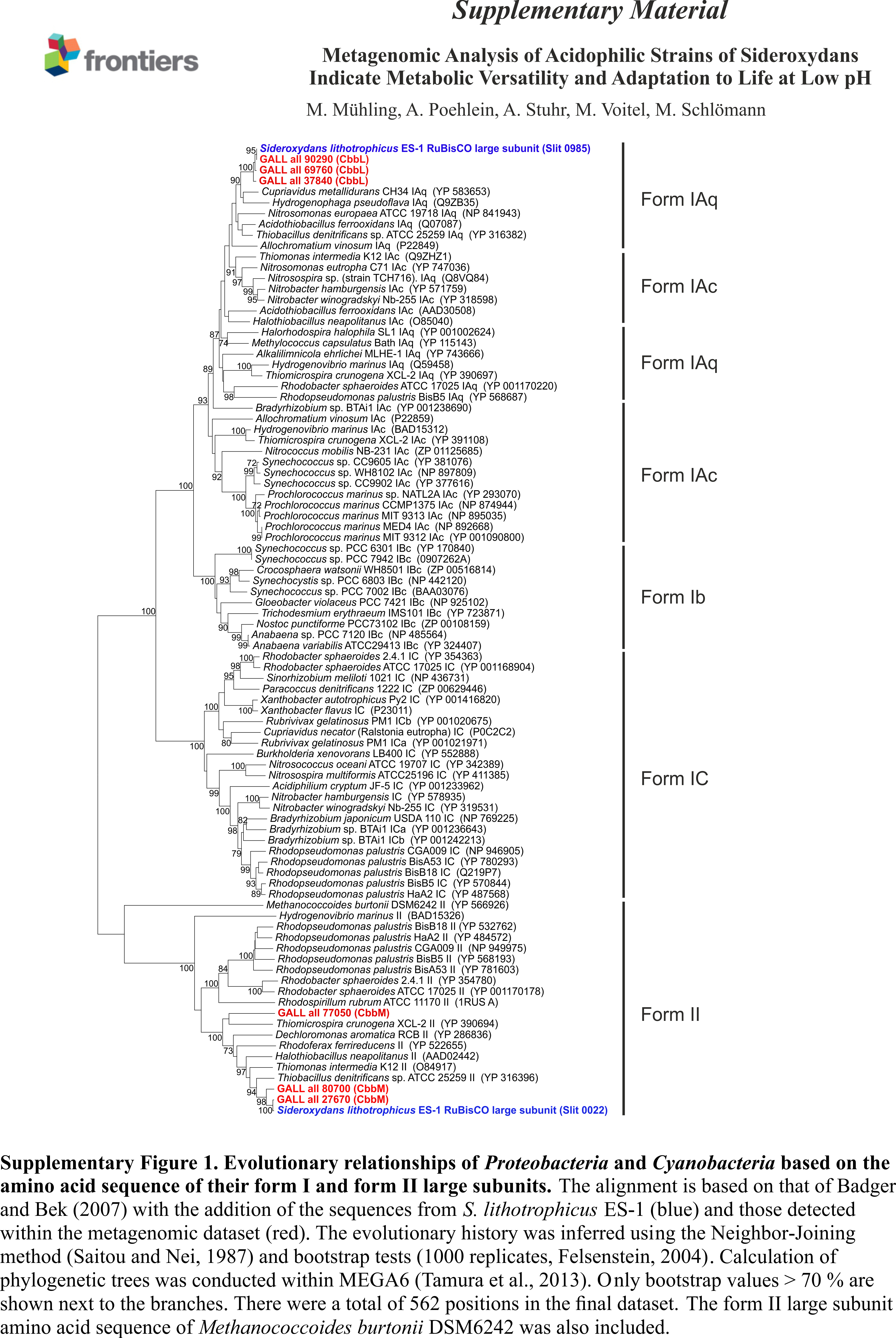

Supplement: Supplementary file 5 [file Image1.jpg]

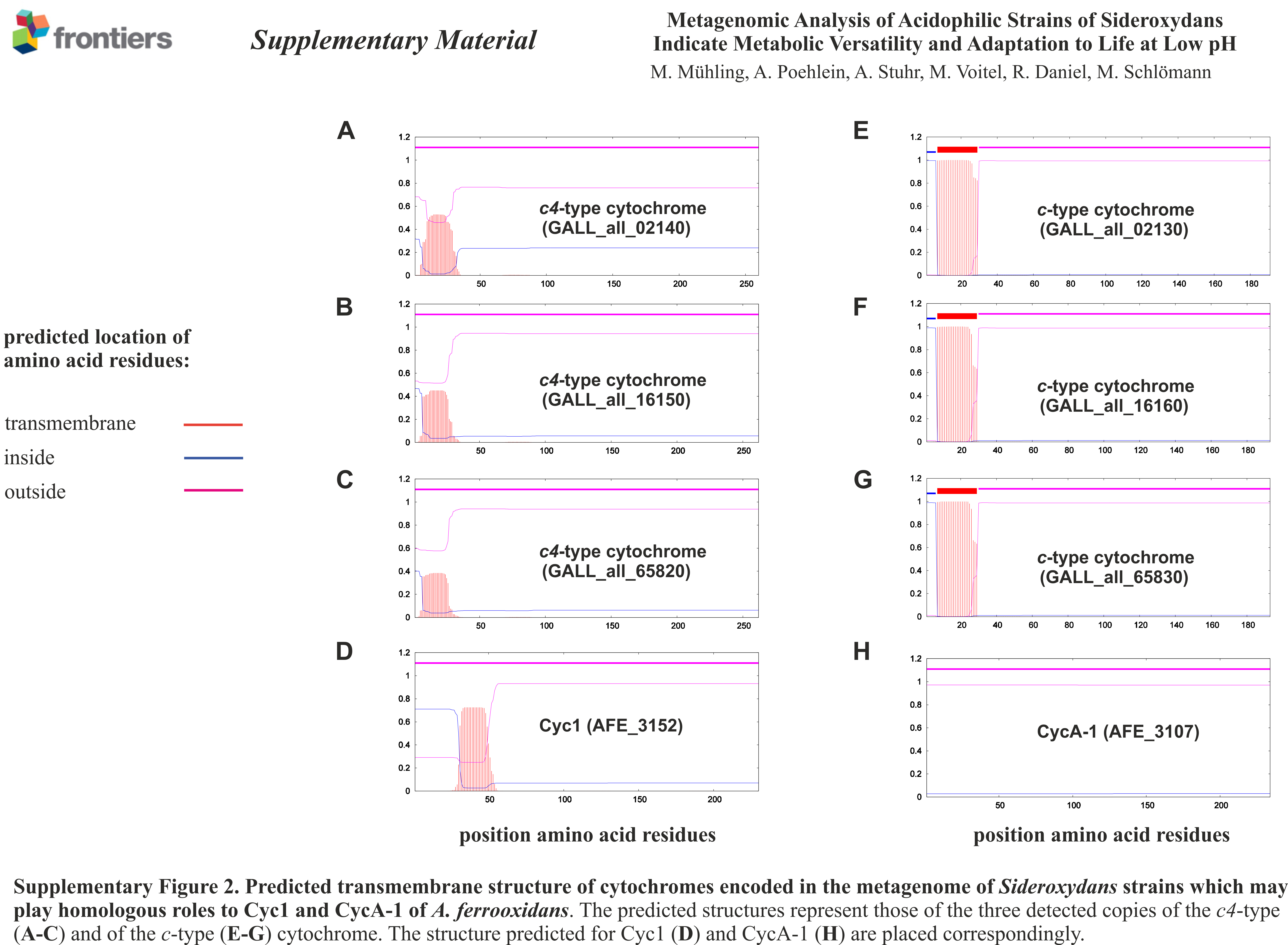

Supplement: Supplementary file 6 [file Image2.jpg]
